# Fostering better policy adoption and inter-disciplinary communication in healthcare: A qualitative analysis of practicing physicians’ common interests

**DOI:** 10.1371/journal.pone.0172865

**Published:** 2017-02-24

**Authors:** Eric J. Keller, Megan Crowley-Matoka, Jeremy D. Collins, Howard B. Chrisman, Magdy P. Milad, Robert L. Vogelzang

**Affiliations:** 1 Center for Bioethics and Medical Humanities, Northwestern University Feinberg School of Medicine, Chicago, Illinois, United States of America; 2 Department of Radiology, Northwestern University Feinberg School of Medicine, Chicago, Illinois, United States of America; 3 Department of Obstetrics and Gynecology-Reproductive Endocrinology & Infertility, Northwestern University Feinberg School of Medicine, Chicago, Illinois, United States of America; IRCCS Istituto Auxologico Italiano, ITALY

## Abstract

**Purpose:**

In response to limited physician adoption of various healthcare initiatives, we sought to propose and assess a novel approach to policy development where one first characterizes diverse physician groups’ common interests, using a medical student and constructivist grounded theory.

**Methods:**

In 6 months, a medical student completed 36 semi-structured interviews with interventional radiologists, gynecologists, and vascular surgeons that were systematically analyzed according to constructivist grounded theory to identifying common themes. Common drivers of clinical decision making and professional values across 3 distinct specialty groups were derived from physicians’ descriptions of their clinical decision making, stories, and concerns.

**Results:**

Common drivers of clinical decision making included patient preference/benefit, experience, reimbursement, busyness/volume, and referral networks. Common values included honesty, trustworthiness, loyalty, humble service, compassion and perseverance, and practical wisdom. Although personal gains were perceived as important interests, such values were easily sacrificed for the good of patients or other non-financial interests. This balance was largely dependent on the incentives and security provided by physicians’ environments.

**Conclusions:**

Using a medical student interviewer and constructivist grounded theory is a feasible means of collecting rich qualitative data to guide policy development. Healthcare administrators and medical educators should consider incorporating this methodology early in policy development to anticipate how value differences between physician groups will influence their acceptance of policies and other broad healthcare initiatives.

## Introduction

Fostering professional behavior change is a key step to improve healthcare quality. This can be complicated by many factors, but one major challenge has been developing initiatives and quality metrics that resonate with diverse professionals’ perceptions of value to garner wide-spread adoption. For example, the Centers for Medicaid and Medicare Services’ (CMS) Meaningful Use initiative was recently ended due to a lack of physician support, with CMS administrator Andy Slavitt noting, “we have to get the hearts and minds of physicians back. I think we’ve lost them” [1]. In this article, we propose that much of this challenge is due to a limited understanding of common values of different physician professional groups which may be addressed by incorporating qualitative analyses of professional values into early policy development.

We believe this challenge is largely under-recognized due to the ubiquitous use of vague value language in healthcare reform and limited available descriptions of professional values that drive clinical decision making. Terms such as cost-effective, patient-centered, and evidence-based permeate many key goals and outcomes for healthcare quality, yet these concepts are rarely defined beyond vague descriptions. Instead healthcare “value” is often equated with economic preferences and efficiency while “appropriateness” and “quality” are used as surrogates for adherence to guidelines produced by groups of experts [2, 3]. Descriptions of common professional values are more clearly defined in quality and ethics literature but are often purely economic or altruistic with rare attempts to blend these interests [4, 5]. Thus, when carefully-formulated guidelines fail to garner and/or sustain adoption and adherence, it is often assumed that physicians must be mis-interpreting available data or pursuing ulterior, self-serving interests [6–8]. This is likely part of the reason why many initiatives aimed at changing physician behavior now use financial incentives, an approach that will be expanded by the 2015 Medicare Access and CHIP Reauthorization Act.

Alternatively, past anthropological and management literature has highlighted the complexity of professional values and the importance of understanding this complexity to develop shared goals that motivate behavior change among diverse groups of professionals. Although financial incentives can be effective, they often fail to motivate complex actions and may oversimplify physicians’ motivations [9, 10]–it is one thing to pay someone to check an A1c level, another to reward “appropriate care” of an individual. Professionals are also driven by autonomy, mastery, and a sense of purpose, and if a system incentivizes actions that undermine these values, it can “sap” physicians of their internal drive [9, 11, 12]. Additionally, physicians are not a monocultural group. Underappreciated values specific to professional groups can cause tension and undermine efforts to promote shared goals and institution-wide initiatives [13, 14]. Such cultural communication barriers have been described among hepatologists and transplant surgeons [15], gynecologic sub-specialties [16], internal medicine services and consulting specialists [17], and physicians and healthcare administrators [13]. Thus, it has been proposed that one must acknowledge and support unique groups’ professional identities while finding their common interests in order to foster shared goals and behavior change [13, 18], i.e., it is not a lack of virtue or interest that limits physicians’ adoption of guidelines but the range of values and experiences they possess [19].

In light of this previous research, we sought to develop a model for healthcare policy development, where one first characterizes the range of stakeholders’ values related to a service and then works inductively to develop and promote initiatives/metrics. Our division of interventional radiology (IR) was interested in better understanding a professional conflict with gynecologists (OBGs) over the proper treatment of systematic uterine fibroids, so we chose to investigate the relationships between IRs and OBGs. We also chose to include vascular surgeons (VSs) whose scope of practice overlaps to a significant degree with IR but does not overlap with OBG. We believed this would serve as an appropriate case example because IRs create novel treatments for conditions managed by other specialties and are also heavily reliant on referrals. We believe this makes them prone to inter-group conflicts with other medical specialty groups.

In order to characterize common interests that drive these professional groups’ (IR, OBG, VS) clinical decision making, we relied on a well-validated method from the social sciences called “constructivist grounded theory” (C-GT). Previous anthropological studies exploring patient and physician values have used GT as a more sensitive qualitative method than surveys that are often used in healthcare to assess value [15, 16, 20, 21]. C-GT is one form of this method that is unique in not assuming that researchers approach the investigation with a completely blank slate and omitting some analysis steps after main results become apparent [22, 23]. A key advantage of GT is the ability to achieve a rich qualitative analysis with relatively small sample sizes, obtaining the majority of information from relatively homogenous groups in the first 6 interviews and almost all new information in the first 12 [24]. We found this method intriguing, but physicians are not an easy group to interview without getting formal filtered answers, and the method’s sensitivity comes with a price of increased risk of bias from the interviewer(s) and data analyzer(s). We believed that medical students are well-situated to serve as physician interviewers. As new members of the larger physician community, students would not be perceived as threatening; and because they are learners, physicians might be inclined to support them by participating in their research. Thus we used a medical student mentored by an experienced medical anthropologist for data collection and analysis, and hypothesized that despite differences in IRs’, OBGs’, and VSs’ approaches to patient care, one could identify common interests and concerns by listening carefully to how they framed their concerns and clinical reasoning.

## Methods

This study was reviewed and approved by a Northwestern University Institutional Review Board (STU: 00105347). All participants were told that their participation would be kept confidential. Written or verbal informed consent was obtained and documented for in-person and phone interviews, respectively, as instructed by the IRB.

### Medical student training

Prior to data collection, the medical student interviewer (EK) met with the experienced medical anthropologist (MC) to discuss the methodology and common pitfalls. The student was recommended texts that were read prior to the first interview [22, 25]. After the first 5 interviews were completed and transcribed, the student, a physician reviewer (RV), and the medical anthropologist analyzed 3 transcripts separately and then met to provide feedback on the interviewing and analysis techniques. This was repeated with an additional 2 transcripts after 50% of the data had been collected. The student used this project as part of his thesis in medical humanities and bioethics and spent considerable time reflecting on the experience while preparing his thesis.

### Interview methods

One Northwestern Medicine (NM) physician in each specialty was chosen at random for the first interview. Subsequent interviews were generated by asking interviewees to recommend colleagues that might offer unique perspectives to reduce sampling bias and collect the broadest possible range of views. A minority (<25%) of participants were asked to recommend colleagues with specific demographics in order to have cohorts’ gender ratios match those of the entire specialty groups and include a mix of practice environments (academic v. private practice) and years of experience. Interviews were initially limited to the Chicago metropolitan area to ensure consistent interview themes were identified before allowing physicians to recommend colleagues from other cities. See Table 1 for interviewee demographics.

**Table 1:** Physician interview demographics.

	IR (n = 12)	VS (n = 12)	OBG (n = 12)
Gender (M / F)	10 / 2	10 / 2	6 / 6
Environment (Academic / Private Practice)	6 / 6	9 / 3	6 / 6
Median Years Post-training (Range)	11 (1–31)	18 (3–33)	21 (5–36)
Chicago / Non-Chicago*	8 / 4	8 / 4	10 / 2

*Physicians were included from California, Arkansas, Ohio, Wisconsin, and North Carolina

Interviews were conducted by the medical student in a semi-structured fashion to adjust for emerging themes and facilitate a conversational tone [26]. See Table 2 for example interview outline. Each physician was asked to describe his/her practice, an interaction with a typical patient, his/her treatment of uterine fibroids (IRs and OBGs) and/or endovascular work (IRs and VSs), how other physicians within and outside his/her specialty treat these patients, how he/she felt about any perceived differences in treatment, sources/solutions to any expressed concerns, and follow up questions for additional detail.

**Table 2:** Example interview outline.

“To start, could you tell me about your practice, the kind of patients you see and any other roles?”
“What led you to be a [physician’s specialty]?” “What about [physician’s specialty] attracted you?”
“Can you take me through a typical patient interaction, how that conversation goes, how you decide what to do next?”
“What factors into that decision?”
“Do other [physician’s specialty] approach [condition/patient population] that way?” “Why/why not?”
“What other specialties that treat [condition/patient population], do they approach it the same way?” “Why/why not?”
“What do you think about those differences?” “Are they good, neutral, concerning to you?” “Why/why not?”
“What could be done about [voiced concern/issue]?”
“Is there a healthcare system or environment that would make that better?

### Qualitative analysis

Interviews were recorded, transcribed verbatim, and analyzed according to constructivist GT [22], using NVivo 10 (QSR International). Key concepts that emerged in the first interviews included physician identities (us versus them), “my patients”, treatment value, physician interests and concerns about physician interests, and concerns regarding physician environments. Additional interviews allowed these concepts to be further defined and compared within and between specialties to identify larger themes and refine conclusions.

All transcripts were analyzed by the student (EK) and reviewed separately by a physician (RV). These researchers met regularly to discuss identified concepts and themes, resolve any discrepancies in their interpretation of the interviews, and reflect upon how their views and experiences may have influenced the analysis. We noted immediately that each specialty had unique ways of describing physician roles, patients, and values. Thus, we found that common drivers and values were best derived not only from those interests explicitly named by physicians but also those identified from carefully analyzing their unique examples, stories, and concerns. To both validate and add to these results, identified drivers/values were compared to previous descriptions of medical virtue ethics [27–29], business ethics [30, 31], medical professionalism [32, 33], medical business ethics [4, 34–37], and historic ethical codes [38–45].

## Results

### Method feasibility

Interviews were completed over approximately 6 months. Most physicians were very open to speaking with the student and sharing their experiences and opinions. Due to the student’s inexperience with this technique, the first 2–3 interviews were less smooth and conversational. Nevertheless, all interviews provided similar quality data. Discrepancies in identified themes among reviewers were rare (2–3 occurrences), requiring discussion with the medical anthropologist to come to a consensus.

The medical student researcher found the training, data collection, and data analysis to be manageable during his medical school curriculum. As a result of the experience, he was able to identify mentors in these specialties and reflect on how he could use the experience to better communicate with his colleagues as a future physician.

### Specialty-specific views

Common themes emerged within the first 3 interviews among IRs and VSs, and among the first 6 interviews for OBGs. Each specialty had unique means of describing their roles and approaches to patient care that remained remarkably constant across practice reimbursement structures and locations. For example, IRs highly valued and defined their practices by the minimally-invasive procedures they offered. Thus, their descriptions of the value of different treatment options often focused on comparing treatments’ invasiveness. They also perceived differences in specialties’ clinical decision making as the result of specialists prioritizing the procedures they themselves offered. This was distinct from VSs and OBGs who defined their roles by the diseases and patient populations they treat, respectively.

### Common physician drivers

**Fig 1:**
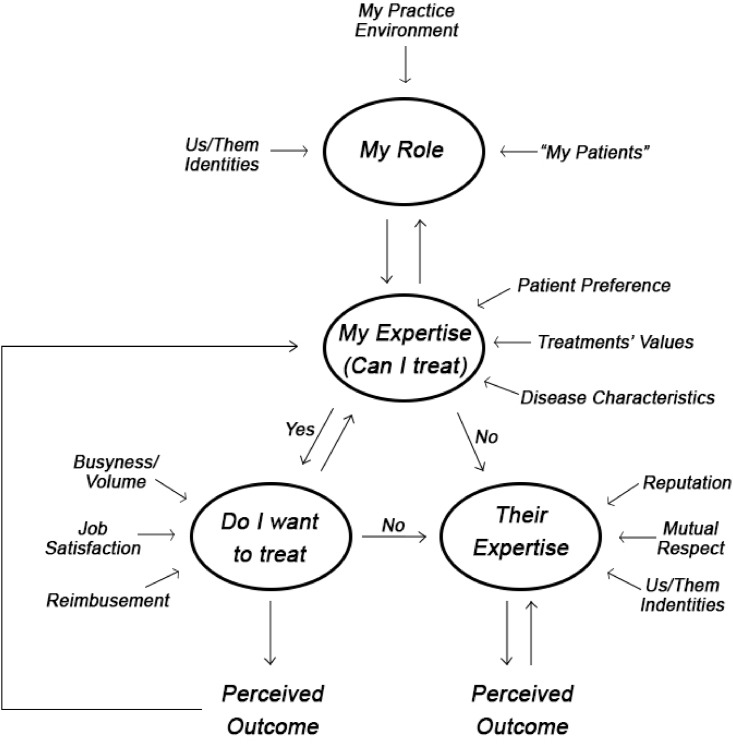
Summary of interviewed physicians’ decision-making framework. Visual representation of common elements of interviewed physicians’ decision-making frameworks. Circles represent critical aspects of clinical decision making which can be more or less influenced by the surrounding factors. The flow is circular, feeding back upon itself to represent the heavy reliance of clinical decision making on past experiences.

The six most commonly described drivers of clinical decision making across these three medical specialties were patient preference, patient benefit, expertise/experience, reimbursement, busyness/volume, and referral networks. See Table 3 for example quotes for each of these themes. All interviewed physicians expressed a strong interest in either “doing right *by* or *for* the patient,” either emphasizing guiding patients through treatment options and patient satisfaction or a balance between patient autonomy and what the physician felt was best. All interviewees described perceived expertise or “tools in your toolbox” as a major driver of behavior, relating not only to who should treat the patient but also defining one’s professional role. Some felt this was the strongest driver of clinical behavior. Reimbursement was described by 31/36 interviewees as significantly driving physician behavior, though this was often ascribed to others. 27/36 physicians mentioned busyness/volume, often positively as a measure of a practice’s success or more neutrally as “hunger” linked to gaining expertise or reimbursement and making it easier to act altruistically if one’s hunger was satisfied. Referral networks were also discussed in 34/36 interviews often in terms of maximizing patient benefit/satisfaction or as key professional relationships allowing one to have a successful and secure practice. Research and education of trainees were also occasionally mentioned, but did not seem to affect decision making as significantly. See Fig 1 for decision tree.

**Table 3:** Example quotes of physician drivers.

**Patient Preference**	**“I CAN REALLY FOCUS ON THESE ARE WHAT YOUR ISSUES ARE… LET’S TALK ABOUT WHAT THE PROS AND CONS ARE FOR YOU AND THEN I ALWAYS END IT WITH SAYING, YOU KNOW, SO WHAT ARE YOU THINKING, WHAT DO YOU WANT TO DO, WHAT ARE YOU MOST INTERESTED IN?” (OBG #2)**
**Patient Benefit**	“I lay the decisions out, but I will give them my opinion. I think a physician has an ethical responsibility to be a doctor, right? Patients come to you for an opinion……and I express it as such.” (IR #3)
**Experience**	“I would say that most of the time largely in the medical community everywhere, including in the community it’s not about evidence-based. It’s about we can do it, and we can do it safely. That simple.” (IR #1) “…we all have our areas of expertise and for any of us to say, I counsel my patients fairly and I tell them about all of their options, it sounds good on paper but, you know, we really aren’t in a position to do that because like I said, medicine is so subspecialized… it’s not a matter of being smarter or less smart.” (OBG #5)
**Reimbursement**	“…ultimately I hate to say this but it generally boils down to money, it’s the dirty little secret of medicine that money really matters…” (VS #4) “Yeah it is something that is basic in medicine and often not talked about, which is the primary driver for a lot of practices is monetary; that is what it is a business. I think that affects clinical decision-making far too often.” (OBG #7)
**Busyness/Volume**	“There are certain places where people may start out and they’re going to be very hungry and so they’re gonna look for anything and everything that they can do for that falls under their area of training…” (IR #2)
**Referral Network**	“…we are our own subspecialty we do rely on a lot of referrals, so you don’t want to step on other people’s toes but at the same time I do operative procedures and I could refer them back… for these same operative procedures too so you have to sort of strike a balance.” (OBG #10)

### Common virtues

Interviewees across specialties tended to feel that better healthcare requires both the right “environments” and “personalities”, i.e., the “soul/presence” of an institution is important, but “at the end of the day… we know right from wrong” (OBG #5). Individually, many physicians stressed the importance of promoting “professionalism/excellence”–the general idea being “…do the right thing and patients will come,” (VS #11) that patients pick up on physicians’ values. After comparing interview themes across specialties to past work, we arrived at six primary physician virtues, summarized in Table 4 with example quotes and described below.

**Table 4:** Summary of common physician virtues.

**Honesty (Know Thyself)**	**BE HONEST WITH ONESELF ABOUT ONE’S EXPERTISE, LIMITS, AND MOTIVATIONS AND CONVEY THIS TO PATIENTS BY ADMITTING BIAS, EXPERIENCE, AND OTHER LOYALTIES/COMMITMENTS**. **“IT HAS ALWAYS BEEN MY PERSPECTIVE AND I THINK I HAVE SEEN IT COME TO FRUITION IS THAT HONESTY FACTOR. YOU HAVE TO TELL THE PATIENT WHERE YOUR COMFORT ZONE IS. IF YOU THINK SOMETHING IS GOING TO VERY CHALLENGING AND DIFFICULT, I THINK YOU HAVE TO TELL THEM. YOU CAN ALSO BE HONEST AND SAY YOUR SURGERY IS GOING TO BE DIFFICULT, SO I’M GOING TO HAVE ANOTHER SURGEON AT THE TABLE, MAYBE YOU WANT THEM TO MEET THEM….” (OBG #7)**.
**Trustworthiness**	Convey openness, mindfulness, and respect to patients, colleagues, and other team members. “I spend 30 minutes for a new patient, he spends 15 minutes for a new patient and I always tell him, you cannot win the trust in 15 minutes, you know, you may make less money but they need to trust you.” (VS #12)
**Loyalty**	Be committed to maintaining strong relationships based on the previous virtue, but be wary that multiple loyalties must be prioritized and clearly expressed as such. “I've maintained my relationships with them so I should probably disclose early on that [IR #3] has my cell phone and I have his cell phone and we see patients on Tuesday mornings at the same time. It’s very common for him to send a patient, I always deliberately like no matter what I'm doing will squeeze them in somehow because I want to foster that relationship. I think it’s so important to be able to maintain that kind of comradery.” (OBG #1)
**Humble Service**	Convey modesty, humility, and temperance with one’s position of wealth and/or power. Seek fair compensation while placing the good of the patient and justice above pursuit of excess personal gains. “…I like guitars and I'm selfish, but I'm very sensitive to people… some of these cancer patients I can’t get them better, but the one thing that I can do is show them some respect, and I can’t tell you how many times family members have stopped by and said “my mom passed away last month but I just want to tell you how much she really appreciated when you took care of her.” It makes me cry; it’s why we do it. Why else would we do it, so we can buy a car, a phone. I mean, it doesn’t matter….” (IR #9)
**Compassion & Perseverance**	Have a tender heart to the needs/concerns of patients while conveying the strength necessary to guide and support patients in the face of death and disease. “I've really stuck to my guns. Once you start compromising your principles then it starts getting a little murky… I'm okay with making less money in life but feeling that I have a good reputation and the way you end up knowing it is when the referring physicians’ family members start coming to you because they know who is very aggressive, who is very conservative, and who is just the right amount of combination between aggressive and conservative.” (VS #12)
**Seek Practical Wisdom**	Develop the wisdom of knowing what options/expertise are available to patients and seek continual feedback and learning to know the best course of action in a given situation, i.e., expertise with context. “There are certain hysterectomies I can do with my eyes shut, and there are cases that may be beyond my skills. Have I been fooled and got into cases that were very challenging and I didn’t project it. If you want to maintain your skills and you have challenging cases, then you do them with somebody who can mentor you and learn from those experiences.” (OBG #8)

“Honesty (Know Thyself)”: The most commonly discussed concern was an unconscious failure “to do the right thing;” and so, interviewees described the need for an “inner voice” or being “honest with yourself” regarding limits and expertise. This failure was often felt to be rampant in medicine and described as physicians only offering what was in their toolbox, failing to incorporate new techniques, or failing to ensure the best person treated the patient. Quotes throughout history have emphasized this value and today it remains emphasized in business ethics [31], medical virtue ethics as “intellectual honesty” [29], and medical professionalism across cultures [32]. Conversely, the failure to know oneself has been described previously in psychology as the “Dunning-Kruger effect”—many people are unaware of what they do not know, and thus it can be difficult to identity one’s own deficits [46].

“Trustworthiness”: Most interviewees described their positive relationships with patients and other physicians in terms of mutual respect, trust, and openness. For example, referral relationships were often described as trusting other physicians to provide superior treatment and conveying mutual respect to patients and/or colleagues in how they received and responded to referrals. To build strong relationships with patients and colleagues, many interviewees expressed the need for openness and respect, whereas inter-specialty turf wars were thought to stem from a perceived lack of these values. This need for openness and trust has long been emphasized in healthcare [33] as well as business [31].

“Loyalty”: This was a strong, positive and constant theme throughout our interviews. Many physicians emphasized their loyalty to their patients’ welfare, their specialty, and/or their practice; whereas unquestioned loyalty or disloyalty in any context were viewed as destructive. In business ethics, there is often discussion of the value of loyalty to one’s stakeholders which has previously been applied to healthcare [30, 31, 35]. Patients are arguably the most vulnerable participants in healthcare, and thus it falls on both physicians and healthcare organizations to ensure that patients’ voices are protected and respected. This prioritized loyalty to patient welfare has long been emphasized in medical ethics as the physician’s fiduciary duty or more recently as patient-centered care [27, 33]. However, loyalty to patients’ welfare does not exclude the value of loyalty to other groups in healthcare (e.g. one’s colleagues or healthcare system) so long as these loyalties are prioritized and such priorities are made clear.

“Humble Service”: Our interviewees often mentioned their commitment to serving their patients even if it meant less economic gain, but many also described complicating factors such as struggling to pay back loans or tension with administration over population-focused vs. individual-focused care. As such, there was significant concern about other physicians consciously pursuing ulterior motives at the expense of patient care (commission)–commonly economic gain (21/36), ego/gaining expertise (11/36), and/or increasing patient volume (11/36)–or actively choosing to not look outside one’s toolbox (omission). This tension is well addressed by the ancient Greek and Catholic virtues of justice and temperance [47]. Most believe that physicians should humbly and selflessly prioritize their patients’ welfare, but treatments providing the maximum benefit are not always the most appropriate use of limited resources [37], and commitment to the welfare of an individual should not necessarily exclude attention to economically responsible care or even seeking *fair* versus *excess* compensation for oneself and/or one’s family.

“Compassion & Perseverance”: Many interviewees expressed balancing sensitivity to patients’ preferences with their expertise/opinion to maximize patient benefit. Some felt strongly that patients and referring physicians “don’t want a wishy-washy physician,” that people come to physicians for their expertise. This balance was particularly promoted by 18^th^ century physician John Gregory, inspiring both Thomas Percival’s medical ethics and the AMA’s first code of ethics. Gregory taught that medicine required sympathy properly expressed in tenderness and steadiness [48]. Absence of either can be problematic, i.e., compassion without perseverance (always do whatever the patient wants) or perseverance without compassion (paternalistic).

“Practical Wisdom”: Experience/expertise was highly valued by all interviewees, whereas failing to seek opportunities to be informed or improve one’s expertise was thought to cause physicians to offer inferior treatments. Often it was felt that physicians should be informed of what their colleagues can offer and share this with patients by offering second opinions and ensuring patients are fully informed of *all* their options. This ideal of practical wisdom gained through experience which allows one to act virtuously in a given situation is the common definition of Aristotle’s “phronesis,” often translated as “prudence”. The importance of seeking prudence has long been emphasized in medicine and has been a central tenet in medical virtue ethics [27].

### The right environment

Beyond the qualities of physicians, interviewees also shared some common views of “better systems.” The specifics varied considerably from single payer systems to more physician control, but two persistent themes were the need for better collaboration and leadership. Because of the difficulty of honestly assessing one’s own performance, many physicians felt there should be administrative delegation of who should be performing which procedures. However, there was also concern about feeling powerless to and misunderstood by leadership. Ideally an administration should be a good coach, modeling professional medical values in critical actions [37], establishing a collective vision or moral tone for the organization [30, 37], and promoting intra-organizational collaboration. As put by a few IRs, it should be Bears v. Vikings not quarterbacks v. linemen (i.e. healthcare organizations competing not physician groups within the same organization). Common examples included aligning economic incentives with other values, encouraging shared goals, clarify roles, creating disease-based, multidisciplinary practices for complex conditions, promoting the goal of relative value units (RVUs) for the system not an individual’s volume, and continuously assessing and improving healthcare quality.

## Discussion

Through this pilot investigation, we sought to assess the feasibility of a novel approach to healthcare policy development using a medical student and constructivist grounded theory to characterize the common interests driving clinical decision making among three distinct professional groups, in this case, 3 distinct medical specialties. In 6 months, we were able to achieve a richer understanding of these groups’ unique and common professional values that could be used to foster better collaboration and develop policies that better reflect these clinicians' senses of value.

Many quality improvement initiatives are limited by poor acceptance among those they aim to affect. This may be due to their theoretical formation often within a single professional group (e.g. medical specialty or administrative organization). For example, the American College of Radiology (ACR) recently received a large grant from CMS to promote collaboration and care coordination between radiologists and referring physicians. The project intends to enroll 4,000 radiologists who will each recruit 5 referring physicians (20,000 in total). Ordering patterns will then be collected and compared to ACR’s appropriateness criteria before and after “educat[ing] our referring physicians about CDS and provid[ing] them with access to ACR Select™,” the digital form of ACR’s appropriateness criteria [49]. Although ACR’s appropriateness criteria were carefully researched and constructed, this project assumes non-radiologists share radiologists’ perception of imaging appropriateness and will be open to being educated about ACR’s criteria. It also assumes that these criteria will resonate with referring physicians enough to meaningfully affect their behavior. The findings from our study may alert the ACR, and other professional organizations, of the importance of consulting other medical specialty groups that are expected to be impacted by the ACR criteria.

The power of using a medical student interviewer and constructivist grounded theory to identify values across medical specialties is that it works from the ground up to derive common interests and engage diverse stakeholders. As a profession, it is not surprising that physicians highly value their experiences—a physician cannot necessarily promise a patient technical success but can promise his/her expertise/experience [50]. Professionalism, or excellence for that matter, is not so much a good but a service. During medical education, trainees undergo intense socialization processes that cause them to identify more as surgeons, for example, than physicians. These divisions can be intensified and charged by the length, difficulty, and isolated nature of their training [21, 51]. With their own training programs, certifying colleges, journals, conferences, and even work spaces, it is not surprising that key experiences driving physician behavior would diverge and cause even two well-meaning physicians from distinct specialties to have significantly different opinions about what the “right” action entails.

By first focusing on common values rather than specific actions, we may garner more adoption of important initiatives. The diversity of specialists’ training experiences and distinct professional identities illustrated in our interviews can make it difficult to appreciate the common interests behind two very different clinical recommendations. Where our interviewees tended to agree was instead on the values/virtues they hoped to convey to their patients and colleagues. The original meaning of “virtues” in ancient Greek philosophy was “tools” or “excellences” which allow an individual to achieve a particular telos or ultimate end. The central telos of medicine has long been healing persons, and so many past expressions of physicians’ virtues have focused on what theoretical attributes physicians’ should possess to best achieve this central objective. Nevertheless, our interviewees rarely, if ever, used terms such as “patient autonomy,” “beneficence,” “intellectual honesty,” or “temperance” despite sharing these values. It is our hope that the virtues we identified based on physicians’ own language and supported by traditional values will better resonate with a larger population of physicians and provide a common language for healthcare improvement while remaining centered on the primary objective of the profession.

Another advantage of our approach is its potential to expose medical students to various specialties’ unique and common values early in their education and facilitate discussions on how one may approach such differences more or less ethically. Recent work in medical education has underscored the importance of focusing on students’ “professional identity formation” [52–54]. Medical education is an important transformation of self to adopt a professional identity as a competent, compassionate physician. Role-models, guided-reflection, and self-awareness are all critical aspects of this process that can be challenging to facilitate [54]. We believe students would be interested in participating in qualitative research about physicians as it affords opportunities for them to network and find mentors while simultaneously allowing medical educators to facilitate guided-reflection and generate valuable data for policy development. In the case of our study, the medical student found the experience both manageable and one of the most valuable experiences of his medical education to date.

Beyond the feasibility and advantages of this approach to healthcare policy development, this study also illustrated the importance of physicians’ virtues and environments. Although reimbursement was perceived as an important driver of physician behavior, it could easily be sacrificed for a sense of purpose from fulfilling an important societal role or mastery of a difficult skill or concept. Much of this balance seemed to rely heavily on the job security and incentives provided by physicians’ environments. Many interviewees noted that prioritizing their shared altruistic values is far easier with greater job security, e.g., not starving for patients, having a strong referral network, and feeling well compensated and supported by administrators.

If we extrapolate our findings and hold that most physicians are committed to doing what is best for their patients, why would so many of our interviewees believe ulterior motives are prevalent in medicine? We believe there is public perception that medicine as a business is wrong [55] creating a false dichotomy that physicians are either selfless saints or money-driven sinners. When media stories highlight physicians prioritizing selfish gains over patient care [8, 56], there is a common assumption that physicians have lost their commitment to act ethically rather than blaming their environments. Certainly both are important. Consider, for comparison, weight loss initiatives. A person can have access to the best support and resources, but at the end of the day, we acknowledge that part of the motivation for change must come from within. Conversely, we acknowledge that people’s willpowers have limits, but that does not make them weak or inferior. Similarly, in healthcare we may do ourselves a disservice if we promote the message that physicians’ interests in their income or other personal gains are unethical while they are surrounded by opportunities to pursue those interests. No perfect set of regulations or healthcare system will eliminate physicians’ need to hold certain professional virtues, but willpower alone is unlikely to make a significant change for many, not because they are unethical, but because they are human. Instead, we can accept that physicians have many values, but we must also work to create environments that incentivize the values we want them to pursue by carefully considering both their financial and non-financial interests. We believe that engaging physician stakeholders in the development of policies and using common value language will convey respect for their expertise while returning some autonomy that many physicians feel is fleeting in the face of increased third-party control.

This study had several limitations. In order to complete the depth of our analysis with a single medical student interviewer, we limited our pilot investigation to a small subset of physicians primarily in the Chicago metropolitan area. Nevertheless, the themes we identified remained consistent across practice environments and locations, and we attempted to make our sample representative of specialty demographics. In order to reduce selection bias, we relied on interviewee recommendations for subsequent interviews. However, this also could have biased our sample if those recommended represent the range of views in a single social circle rather than the larger specialty community. Finally, we did not observe physicians’ actions and instead relied solely on their perceptions and opinions. We attempted to make our interview questions non-leading and somewhat indirect to assess physicians’ honest experiences. Nevertheless, future observational studies would be necessary to definitely say whether physicians’ accounts were truly reflective of their actions.

## Conclusions

In response to limited physician adoption of various healthcare initiatives, we proposed and assessed a novel approach to policy development where one first characterizes the complexity and range of stakeholders’ professional values and then uses this understanding to inform policy development. As a case example, we used a medical student mentored by an experienced anthropologist and C-GT to understand the common values driving clinical decision making among IRs, OBGs, and VSs despite practice variation and tension. We found this approach to be feasible and promising as it works from the ground up while simultaneously engaging physician stakeholders and providing valuable professional development for the medical student. Beyond the feasibility of this promising method, our interviews illustrated an underappreciated complexity of drivers of physician behavior. Although many physicians were interested in financial gains, these interests were often sacrificed for the good of their patients or other non-financial interests. Clinicians felt that altruistic values were important but often undermined by unsupportive environments that create insecurity. Thus, there is a need to better understand the complexity of physicians’ interests and the effects of their environments on a larger scale. Healthcare administrators and medical educators should also consider working together to have medical students collect valuable data for policy development while providing an invaluable educational experience.
